# Phase Separation of Nucleic Acids: Mechanisms, Properties, and Applications

**DOI:** 10.1002/anie.202523943

**Published:** 2026-02-04

**Authors:** Weixiang Chen, Johann Fritzen, Andreas Walther

**Affiliations:** ^1^ Life‐Like Materials and Systems Department of Chemistry University of Mainz Duesbergweg 10–14 55128 Mainz Germany; ^2^ Max Planck Institute for Polymer Research Ackermannweg 10 55128 Mainz Germany

**Keywords:** DNA Nanotechnology, DNA/RNA Condensate, Phase Separation, Synthetic Biology, Synthetic Cell

## Abstract

Nucleic acids are essential biological macromolecules bearing genetic information and playing important roles in post‐transcriptional regulation. Given their high programmability based on Watson–Crick–Franklin base‐pairing interactions, synthetic DNA and RNA oligonucleotides have become versatile building blocks for programmable assembly of nanostructures, nanomachines, and macroscopic materials. Recent discoveries have shown that long‐chain nucleic acids can undergo temperature‐induced phase separation, enabling rapid and facile formation of micro‐sized, nucleic acid‐rich condensates. Unlike conventional DNA/RNA nanotechnology, which relies primarily on base‐pairing interactions, phase separation leverages the intrinsic polymeric nature of nucleic acids. While it expands the scope of DNA/RNA nanotechnology for new applications, nucleic acid phase separation also provides a fresh perspective for how compartmentalization may have emerged in the prebiotic RNA world during the origin of life. In this Minireview, we discuss the current mechanistic understanding of temperature‐induced phase separation of synthetic long‐chain DNA and RNA in vitro, in the absence of complex coacervation with proteins and polymers. We highlight strategies for controlling the physical and chemical properties of DNA condensates and review the progress and advances in developing them for various applications.

## Graphical abstract

Recent discoveries have shown that single‐stranded long‐chain nucleic acids can undergo temperature‐induced phase separation, enabling formation of micrometer‐sized condensates. This Minireview discusses the current mechanistic understanding of this phenomenon, highlights strategies for controlling the physical and chemical properties of these condensates and summarizes advances in developing them for various applications.

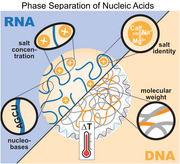

## 1 Introduction

DNA stores genetic information and underpins the central dogma of molecular biology.^[^ ^1^, ^2^, ^3^ ^]^ In the 1980s, Nadrian Seeman proposed repurposing synthetic DNA oligonucleotides as programmable building blocks that self‐assemble via predictable base‐pairing.^[^ ^4^ ^]^ Seeman's pioneering works for DNA junctions, cubes, and double‐crossover tiles initiated a new research direction: DNA nanotechnology.^[^ ^5^, ^6^, ^7^ ^]^ Since then, DNA has become a versatile material across different disciplines. Landmark advances, including DNA origami,^[^ ^8^ ^]^ DNA‐based logic computation and circuits,^[^ ^9^, ^10^ ^]^ DNA crystals,^[^ ^11^ ^]^ and DNA walking devices,^[^ ^12^, ^13^ ^]^ have profoundly shaped DNA nanotechnology research from nanostructures to computation and nanomachines.^[^ ^14^ ^]^

While molecular recognition via base pairing allows programmable nanostructure formation with high precision, such assemblies remain confined to the nanoscale unless hybridized with other components. New interactions were required to extend all‐DNA materials to mesoscopic dimensions. In 2018, we discovered that long single‐stranded DNA (ssDNA) can undergo sequence‐dependent phase separation (PS) at elevated temperatures, yielding micrometer‐sized DNA condensates.^[^ ^15^ ^]^ In 2023, a similar temperature‐induced PS was reported for RNA by Banerjee and coworkers.^[^ ^16^ ^]^ This heating‐induced PS corresponds to a lower critical solution temperature (LCST) phenomenon, with the LCST designating the lowest temperature of the phase diagram and cloud points of varying temperatures (*T*_cp_) being present at each composition that undergoes PS. This LCST behavior, previously well established for many synthetic polymers,^[^ ^17^ ^]^ underscores fundamentally the polymeric nature of nucleic acids. Notably, RNA PS bears strong biological relevance, as transcriptional and RNA–protein condensates form in cells,^[^ ^18^, ^19^, ^20^ ^]^ and may offer insights to prebiotic compartmentalization and the origin of life.^[^ ^21^, ^22^ ^]^

Leveraging LCST‐type PS, we and others have advanced DNA (and, to a lesser extent, RNA) condensates beyond their traditional role as organizer of cellular processes. These efforts have transformed them into synthetic cells (SCs) equipped with engineered properties and capabilities for more complex behaviors and cell‐like functions, including internal secondary structures, prototissue formation (large SC clusters) by co‐assembly of hundreds of SCs, metabolic and signaling activity, and even cellular regulation.^[^ ^15^, ^23^, ^24^, ^25^, ^26^, ^27^, ^28^, ^29^, ^30^ ^]^ The high concentration of nucleic acids in condensates creates a macromolecularly crowded, viscoelastic interior that conceptually mirrors the cytoplasm of living cells.^[^ ^31^, ^32^, ^33^ ^]^ Parallel studies have begun to unravel the mechanisms governing nucleic acid PS.^[^ ^16^, ^34^, ^35^ ^]^ Consequently, DNA and RNA condensates have emerged as a new class of programmable nucleic acid materials with broad potential in synthetic biology and materials science. We note that there are also isothermal approaches for engineering DNA/RNA condensates by DNA/RNA nanostars, which is not the focus of this minireview.^[^ ^36^, ^37^, ^38^, ^39^, ^40^ ^]^

In this Minireview, we summarize the current mechanistic understanding of temperature‐induced PS of synthetic long‐chain nucleic acids in vitro, in the absence of complex coacervation with proteins and polymers, and highlight the unique properties of DNA/RNA condensates as a new class of materials. We outline strategies to control and engineer their behavior and survey emerging applications, emphasizing their role as model systems for SCs. Finally, we discuss future directions for nucleic acid condensate research, including mechanistic studies, their role in SC systems, and their broader impact on DNA and RNA nanotechnology.

## 2 Mechanism for the Temperature‐Induced PS of Nucleic Acids

To elucidate the mechanism of nucleic acid PS, we first outline the structural distinctions between DNA and RNA. We then discuss how intrinsic factors, such as purine versus pyrimidine composition and molecular weight, and extrinsic factors, such as salt type and salt concentration, govern PS behavior. The interplay of these parameters can induce partial or complete percolation transitions, in which intermolecular interactions give rise to a system‐spanning network, thereby tuning condensate dynamics and metastability.

### 2.1 Structures of DNA and RNA—Similarities and Differences

Structurally, DNA and RNA are closely related macromolecules, yet subtle differences make them selectively recognizable in biological systems. Both are linear polymers of phosphodiester‐linked nucleotides. In DNA, the sugar is deoxyribose, whose 1′ position binds one of four nucleobases—adenine (A), cytosine (C), guanine (G), or thymine (T). Phosphodiester bonds connect the 3′‐hydroxyl of one sugar to the 5′‐hydroxyl of the next. Each base forms a specific hydrogen‐bonded pair (A–T, C–G), giving rise to antiparallel complementary strands that assemble into the double helix. RNA differs from DNA in three key aspects: (1) It contains ribose instead of deoxyribose, providing a reactive 2′‐hydroxyl group in each nucleotide. (2) Uracil (U) replaces thymine. (3) RNA rarely forms a perfect double helix, instead, it adopts intramolecular folding and complex 3D structures that can exhibit enzymatic activity. This catalytic potential stems from its folded architecture and the 2′‐OH group, which can act as an internal nucleophile in intramolecular transesterification reactions. Consequently, RNA is more hydrolysis‐prone and less stable than DNA, particularly under alkaline conditions.^[^ ^41^ ^]^ Notably, there is currently a discussion that the 2′‐OH group unique to RNA can substantially modify its PS processes, including temperature responses of single chain compaction, phase behavior, and percolation propensity.^[^ ^42^ ^]^

### 2.2 Temperature‐Induced PS

**Figure 1:**
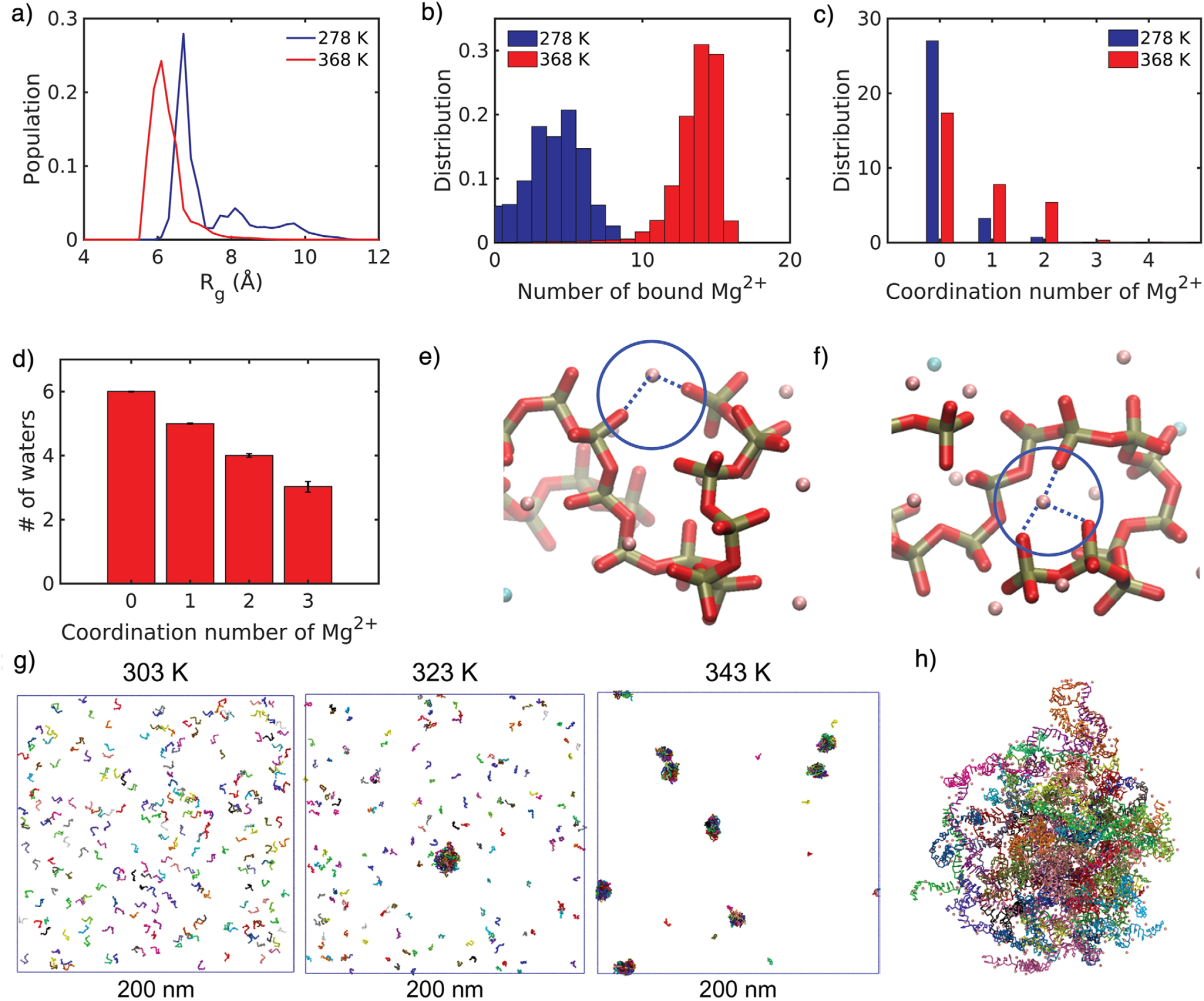
Mg^2+^ bridging induces compaction of poly(P) chains. a) All‐atom MD simulations of poly(P) in the presence of 100 mM MgCl_2_ shows more compact conformations at 368 K than at 278 K. b) Distribution of Mg^2+^ ions bound to poly(P) at 278 K and 368 K and c) the respective coordination number of the Mg^2+^ ions. d) Effect of Mg^2+^ coordination to oxygen on the first solvation shell. e, f) Snapshots of different Mg^2+^ binding modes to two or three oxygen atoms. g) Snapshots of rA_30_ PS at 303, 323, and 343 K in a coarse‐grained model. h) Snapshot of a representative rA_30_ condensate in the coarse‐grained model. Each RNA chain is colored differently, while Mg^2+^ ions are shown as pink beads. Figures a–f are reproduced from ref. [16] with permission. Copyright 2023, The Authors, under exclusive license to Springer Nature Limited. Figures g, h are reproduced from ref. [43] with permission under the terms of the Creative Commons CC BY‐NC‐ND 4.0 license. Copyright 2025, The Authors.

Despite their structural differences, DNA and RNA exhibit comparable behavior in temperature‐induced PS: long strands condense upon increasing temperature and in the presence of divalent cations. The phosphate backbone common to both molecules is the primary driver of this propensity. All‐atom molecular dynamics (MD) simulations of inorganic polyphosphate (poly(P)) revealed that poly(P) adopts more collapsed conformations at higher temperatures in the presence of 100 mM MgCl_2_ (Figure 1a).^[^ ^16^ ^]^ Elevated temperature promotes increased Mg^2+^ binding with higher coordination numbers, leading to intramolecular crosslinking of poly(P) through multivalent interactions (Figure 1b,c,e,f). Backbone desolvation accompanies highly coordinated Mg^2+^ binding (Figure 1d), driving an entropy‐favored collapse into a dense phase. Experiments on temperature‐induced PS of poly(P) corroborate these findings.^[^ ^16^ ^]^ A related coarse‐grained model by Chen and Li confirmed such Mg^2+^‐mediated bridging promoting PS of rA_30_ RNA upon temperature increase (Figure 1g,h).^[^ ^43^ ^]^

### 2.3 Purine‐Rich Sequences Drive PS

**Figure 2:**
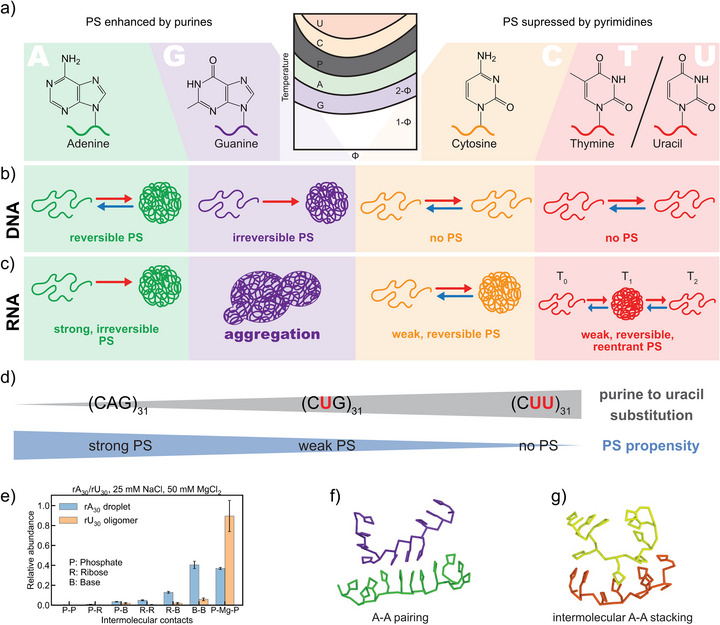
Influence of nucleobases (A, G, C, T, and U) on DNA and RNA PS behavior. a) Molecular structures of DNA and RNA nucleobases and schematic ranking of their PS propensity. b, c) Comparison of nucleobase‐dependent temperature‐induced PS behavior of DNA and RNA. d) PS of (CAG)_31_ in comparison with purine‐to‐uracil variants (CUG)_31_ and (CUU)_31_. e) The relative abundance of various intermolecular contacts within rA_30_ condensates and rU_30_ oligomers. In particular, P‐Mg‐P indicates the Mg^2+^‐bridged backbone phosphate interactions in RNA. f) Representative snapshots of noncanonical A‐A base pairing in a coarse‐grained model. g) Representative snapshots of intermolecular A‐A base stacking in a coarse‐grained model. Figures a, d are adapted from ref. [16] with permission. Copyright 2023, The Authors, under exclusive license to Springer Nature Limited. Figures a‐c are adapted from ref. [15] with permission. Copyright 2018, The Authors. Figures e–g are reproduced from ref. [43] with permission under the terms of the Creative Commons CC BY‐NC‐ND 4.0 license. Copyright 2025, The Authors.

While temperature‐induced backbone desolvation is a common driver of LCST behavior, DNA and RNA exhibit a more complicated PS process that depends on the nucleobase composition. After charge neutralization and entropy‐driven water expulsion, interchain interactions strengthen. The resulting hydrophobic environment promotes base–base interactions such as hydrogen bonding and π–π stacking, that together define the cloud‐point temperature (the point at which PS occurs and the solution becomes visibly cloudy, *T*_cp_). Due to their larger aromatic surfaces, purines exhibit stronger stacking than pyrimidines, yielding lower *T*_cp_ values for purine‐rich sequences (Figure 2a).^[^ ^16^ ^]^ These trends were identified by Walther and coworkers for DNA (Figure 2b),^[^ ^15^ ^]^ and by Banerjee and coworkers for RNA (Figure 2c).^[^ ^16^ ^]^

In agreement with the order of base‐stacking propensity, both nucleic acid types show earlier PS for poly(A) and poly(G). While poly(A) shows reversible PS, poly(G) does not: once backbone desolvation induces stacking, strong interbase interactions, likely reinforced by G‐quadruplex formation, stabilize the condensate even after cooling.^[^ ^44^ ^]^ In contrast, pyrimidine polymers (C, T/U) require higher Mg^2+^ levels for PS, which remains reversible. A remarkable exception is poly(U) RNA, which displays a closed‐loop (LCST < UCST) reentrant behavior. Here, moderate temperatures favor desolvation and base‐stacking‐driven condensation. But at higher temperatures, entropic chain relaxation outweighs the weak enthalpic stabilization, causing redissolution.

Comparative studies of (CAG)_31_, (CUG)_31_, and (CUU)_31_ sequences confirmed that purine‐to‐uracil substitutions increase *T*_cp_, suppressing PS for the double mutant even at 80 °C (Figure 2d).^[^ ^15^ ^]^ Coarse‐grained simulations by Chen and Li further quantified these effects, showing that rU_30_, despite stronger Mg^2+^ bridging, lacks the noncanonical base pairing and intermolecular stacking present in rA_30_, which are essential for PS (Figure 2e–g).^[^ ^43^ ^]^ Overall, while the phosphate backbone drives LCST behavior, purine content enhances PS propensity, whereas pyrimidines suppress it.

### 2.4 Influence of Molecular Weight, Salinity, and Percolation Transition

**Figure 3:**
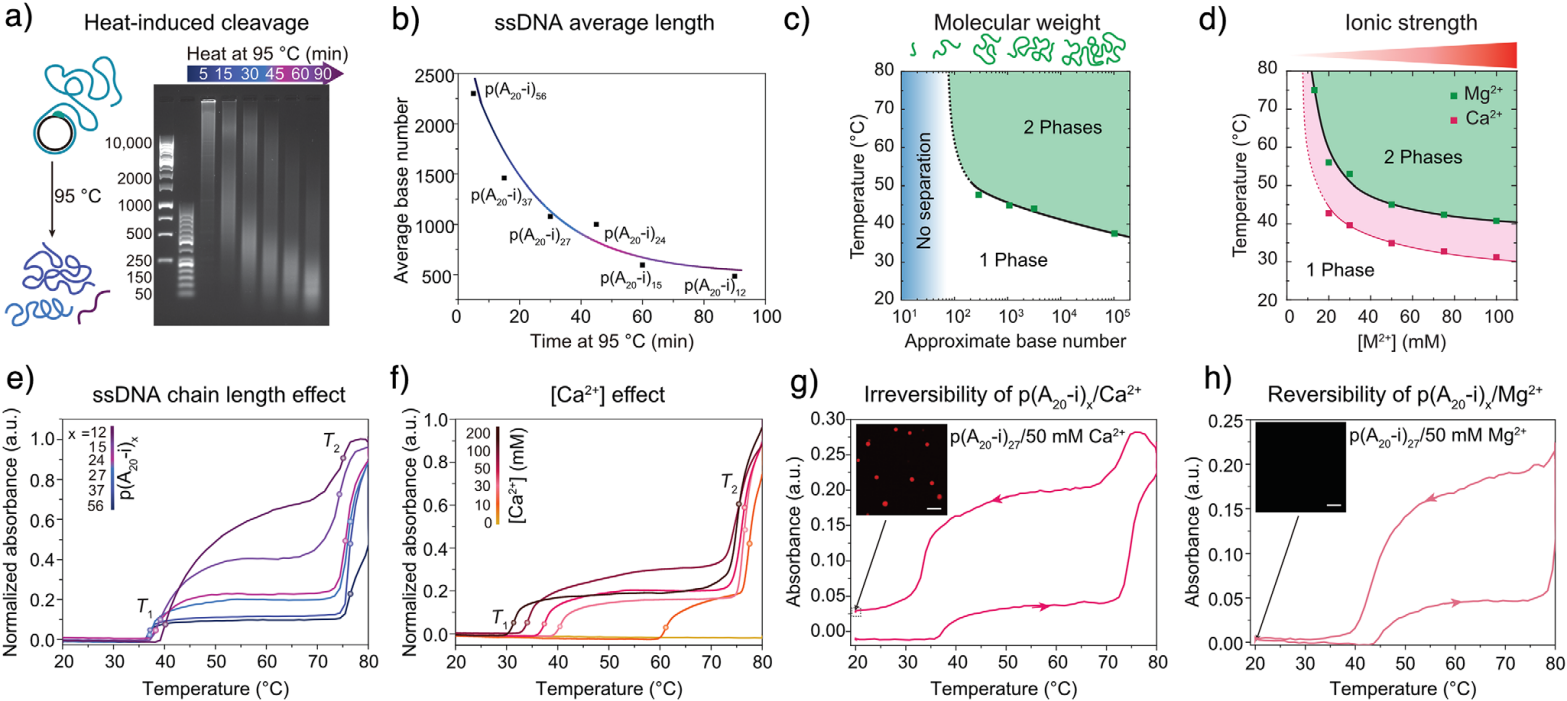
Molecular weight and salt identity determine LCST and reversibility of PS by related percolation transition. a) Agarose gel electrophoresis for the thermal cleavage of poly(A_20_‐i)_n_ (n = repeating units) by heating to 95 °C for different incubation times. b) Average length of poly(A_20_‐i)_n_ as a function of incubation time. c) *T*_cp_ of 0.06 g/L solutions of poly(A_20_‐i)_n_ as a function of molecular weight (in the presence of 50 mM MgAc_2_). Solid and dotted lines are guides to the eye. d) *T*_cp_ of 0.06 g/L solutions of poly(A_20_‐i)_n_ as a function of counterion concentration (MgAc_2_ and CaCl_2_). Solid and dotted lines are guides to the eye. e) Temperature‐dependent absorbance at 350 nm of poly(A_20_‐i)_n_ with different molecular weights in the presence of 50 mM Ca^2+^. f) Temperature‐dependent absorbance at 350 nm of poly(A_20_‐i)_27_ at varying Ca^2+^ concentrations. g) Cyclic temperature ramp showing absorbance at 350 nm of poly(A_20_‐i)_27_ in the presence of 50 mM Ca^2+^. The inset shows a CLSM image after the heating/cooling cycle. h) Cyclic temperature ramp showing absorbance at 350 nm of poly(A_20_‐i)_27_ in the presence of 50 mM Mg^2+^. The inset shows a CLSM image after the heating/cooling cycle. Temperature ramp rate: 1 °C/min. Scale bars are 10 µm for (g) and (h). Figures a, b, e–h are adapted from ref. [34] with permission under the terms of the Creative Commons CC BY‐NC 4.0. Copyright 2022, The Authors. Figures c, d are adapted from ref. [15] with permission. Copyright 2018, The Authors.

The polymeric nature of DNA and RNA is crucial for their LCST behavior, but how does their molecular weight influence PS behavior? Using rolling circle amplification (RCA), very long ssDNA polymers can be generated and shortened by heat‐induced cleavage (Figure 3a,b).^[^ ^34^ ^]^ The *T*_cp_ decreases with increasing chain length, and low‐molecular‐weight ssDNA below 100 nucleobases fails to undergo PS (Figure 3c).^[^ ^15^ ^]^ Phase behavior is also influenced by salt identity and salt concentration (Figure 3d).^[^ ^15^ ^]^ Increasing Mg^2+^ or Ca^2+^ levels lowers *T*_cp_ values by enhancing counterion condensation and reducing electrostatic repulsion. Generally, Ca^2+^ is more effective than Mg^2+^ in promoting DNA PS. Similar trends in the effects of molecular weight and salt concentration are expected for ssRNA, given its structural similarity to DNA and its polyelectrolyte nature. However, quantitative studies are still lacking.

Interestingly, temperature‐dependent turbidity measurements of DNA with varying chain lengths reveal two transition temperatures, *T*_1_ and *T*_2_, observed for both Mg^2+^ and Ca^2+^ (Figure 3e–h).^[^ ^34^ ^]^ Consistent with dynamic light scattering, these correspond to nucleation (*T*_1_) and growth/coalescence (*T*_2_) phases. At constant [Ca^2+^], shorter ssDNA chains exhibit higher turbidity between *T*_1_ and *T*_2_, likely reflecting larger nuclei, while both transition temperatures remain nearly constant. Increasing [Ca^2+^] advances the onset of nucleation (*T*_1_) without changing turbidity magnitude (Figure 3f). Additionally, heating in 50 mM Ca^2+^ produces irreversible PS, with condensates remaining insoluble upon cooling (Figure 3g), whereas 50 mM Mg^2+^ shifts both transitions to higher temperatures and restores the baseline after cooling, confirming reversible PS (Figure 3h). Thus, the divalent cation identity critically determines LCST behavior: both ions induce DNA PS, but Ca^2+^ binding is tighter, rendering the process irreversible, unlike the reversible behavior with Mg^2+^. This also reflects why temperature‐induced PS of nucleic acids has not been observed in conventional polymerase chain reaction (PCR) workflows, where the concentrations of divalent cations (such as Mg^2+^ or Ca^2+^) and of nucleic acids remain relatively low.

### 2.5 PS Coupled to Percolation

**Figure 4:**
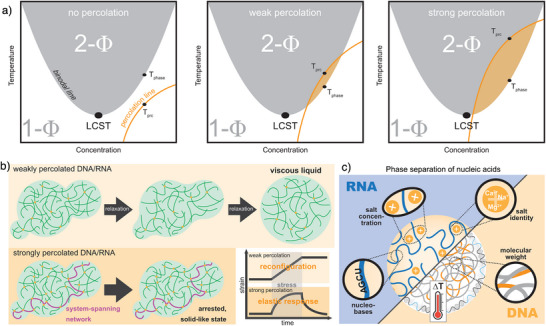
Comparison of weakly and strongly percolated condensates. a) Schematic phase diagrams for nucleic acids with different tendencies of percolation. b) Different chain relaxation and dynamic states for weakly and strongly percolating nucleic acid condensates and idealized representation of stress‐strain behavior of such condensates. c) Summary of influencing factors for temperature‐induced PS of long‐chain, single‐stranded RNA and DNA. Figures are adapted and redrawn from ref. [45] with permission. Copyright 2024, Elsevier Inc.

Failure to redissolve after cooling for specific systems indicates that PS of nucleic acids can lead to system‐spanning network formation, i.e., a percolation transition driven by interchain interactions.^[^ ^45^ ^]^ This is sketched in Figure 4a for different levels of percolation (orange region). Although percolation may occur independently of PS, the two processes often coincide. In nucleic acid condensates, percolation typically arises within the dense phase after PS, and these networks can persist upon cooling. In nonpercolating systems (left diagram of Figure 4a) the two‐phase and percolation regimes do not overlap, so no dense phase exists below the percolation temperature (*T*_prc_). Weakly percolating systems exhibit a small overlap (orange region, middle diagram), where dense‐phase compositions below *T*_prc_ undergo percolation. In strongly percolating systems, this overlap is larger, meaning that many states yield dense‐phase percolation and thus irreversible aggregate formation (right diagram). Weakly percolated condensates can relax toward minimum‐energy shapes through gradual chain rearrangement, whereas strongly percolated condensates become dynamically arrested (Figure 4b). As a result, weakly percolated systems exhibit liquid‐like, reconfigurable behavior under stress, while strongly percolated ones display solid‐like rigidity.^[^ ^45^ ^]^ Notably, such percolation transition applies not only to DNA and RNA condensates, but also to biomolecular condensates in general, as intra‐ and intermolecular interactions are ubiquitous in biological macromolecules.^[^ ^46^ ^]^ Qualitatively, high salt concentrations, high molecular weight, and stronger inter‐chain interactions are expected to promote such percolation transition as they contribute to strengthen the interactions within the dense phase of the formed condensates to form stronger system‐spanning networks.

In summary, due to the polyelectrolyte nature of RNA and DNA, both the PS and percolation transitions are subjected to influencing factors, such as nucleobase composition, salt concentration, salt identity, and molecular weight (Figure 4c).

## 3 Engineering Structures and Dynamics of DNA Condensates

While the mechanistic study of temperature‐induced nucleic acid PS involves both DNA and RNA, engineering efforts for functional molecular systems have focused mainly on DNA. DNA condensates can be tailored in structure and dynamics by varying PS strategies, polymer design, and salinity. With different strategies, membraneless or core–shell condensates can be obtained, featuring tunable localization of defined ssDNA barcodes at core and shell for complementary binding, and adjustable dynamics.

### 3.1 Structure and Barcode Engineering of DNA Condensates

**Figure 5:**
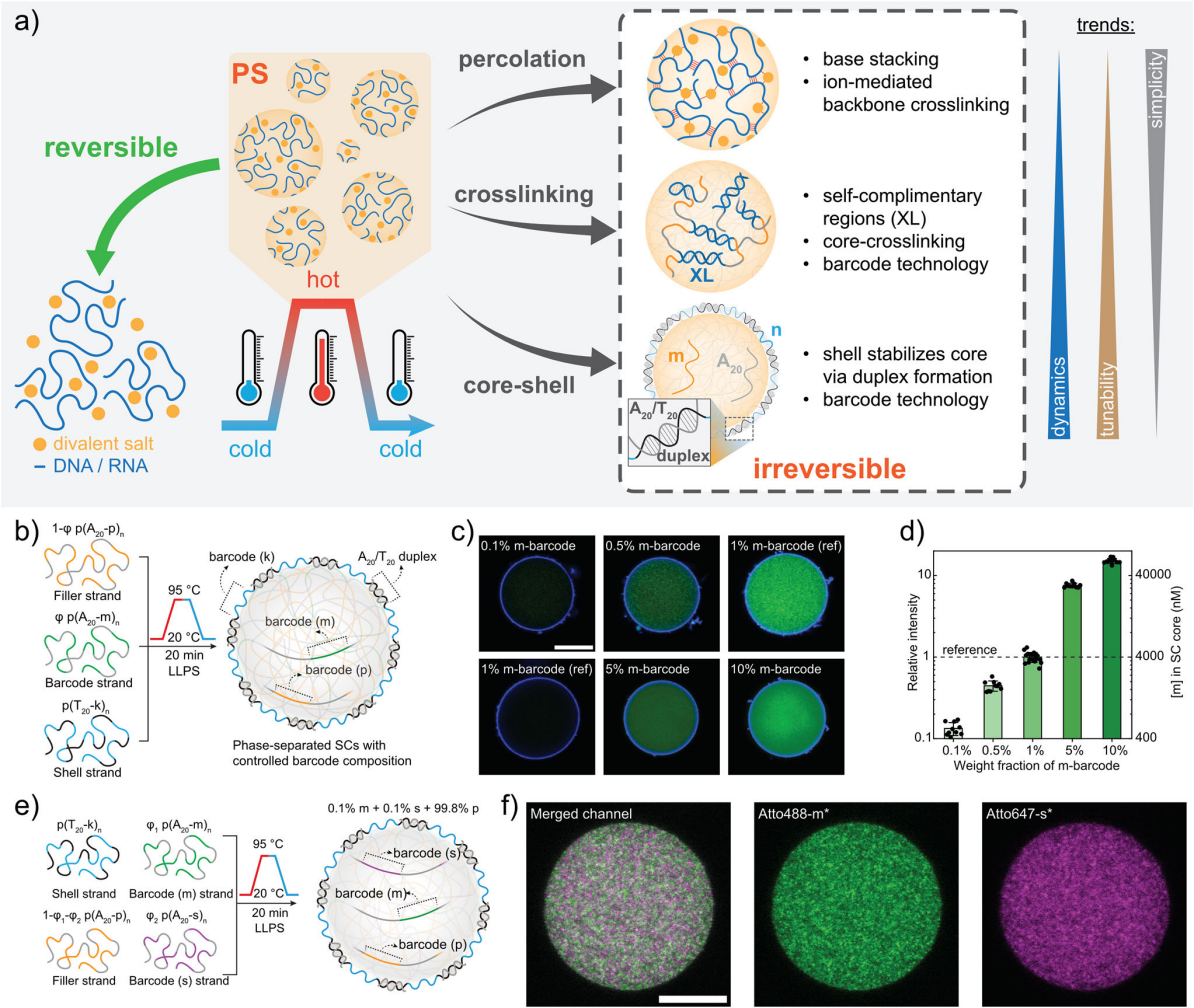
Structure and barcode engineering of DNA condensates. a) Schematic comparison of different approaches to engineering structures based on LCST behavior of nucleic acids. b) Scheme for preparation of condensates containing two barcodes through co‐PS of several ssDNA polymers. c) Representative CLSM images showing the control of m barcode concentration inside condensates, labelled by m*‐Atto488. Note that for each row of the CLSM images, laser settings are the same for direct comparison of internal fluorescence intensity to quantify relative m barcode concentrations ([m]). d) Quantification of [m] inside the condensate core as a function of weight fraction of poly(A_20_‐m)_n_ used during preparation. e) Scheme for the dilution of two barcodes (m and s) at a stoichiometric ratio in a “matrix” of a third poly(A) ssDNA polymer (with barcode p) inside condensates. f) Representative CLSM images showing a condensate containing 0.1% m and 0.1% s barcode, labelled by Atto488‐m* and Atto647‐s*. Scale bars are 10 µm for (c) and (f). Figures b–f are adapted from ref. [27] with permission under the terms of the Creative Commons CC BY license. Copyright 2025, The Authors.

Stabilization of DNA condensates below their *T*_cp_ can be achieved by three main approaches (Figure 5a):

1. Strongly percolated systems—favored for high G/A content, elevated salinity, and Ca^2+^ instead of Mg^2+^—remain stable after heating into the percolation region and cooling below the original *T*_cp_.^[^ ^45^ ^]^
2. Introducing self‐crosslinking palindromic repeats (XL) into phase‐separating ssDNA promotes intra‐ and intermolecular crosslinking upon cooling, stabilizing membraneless condensates with tunable properties.^[^ ^15^ ^]^
3. Heating poly(A)/poly(T) mixtures produces core–shell condensates: poly(A) phase‐separates upon heating, and poly(T) forms a hydrogel shell during cooling via surface hybridization.^[^ ^15^ ^]^ The permeable shell permits diffusion of macromolecules including nucleic acids, synthetic polymers, and enzymes, making these condensates valuable for SC applications.^[^ ^15^, ^23^, ^25^, ^27^, ^28^, ^47^ ^]^

To control condensate composition, we recently demonstrated that multiple poly(A) ssDNA polymers bearing distinct barcode sequences can co‐phase‐separate into a single condensate phase (Figure 5b–f), opening routes for stoichiometric adjustments or dilution of specific barcodes in a “matrix.”^[^ ^27^ ^]^

### 3.2 Engineering of Condensate Dynamics and Related Transport Phenomenon

Depending on composition (sequence, length), environmental conditions (salt type, salt concentration), and PS parameters (molecule concentration, temperature), condensates formed after heating can adopt distinct dynamic states. We take core–shell DNA condensates, prepared via temperature‐induced PS of poly(A) and poly(T) mixtures as an example, which is of highest relevance to SC research and among the most deeply investigated systems.

**Figure 6:**
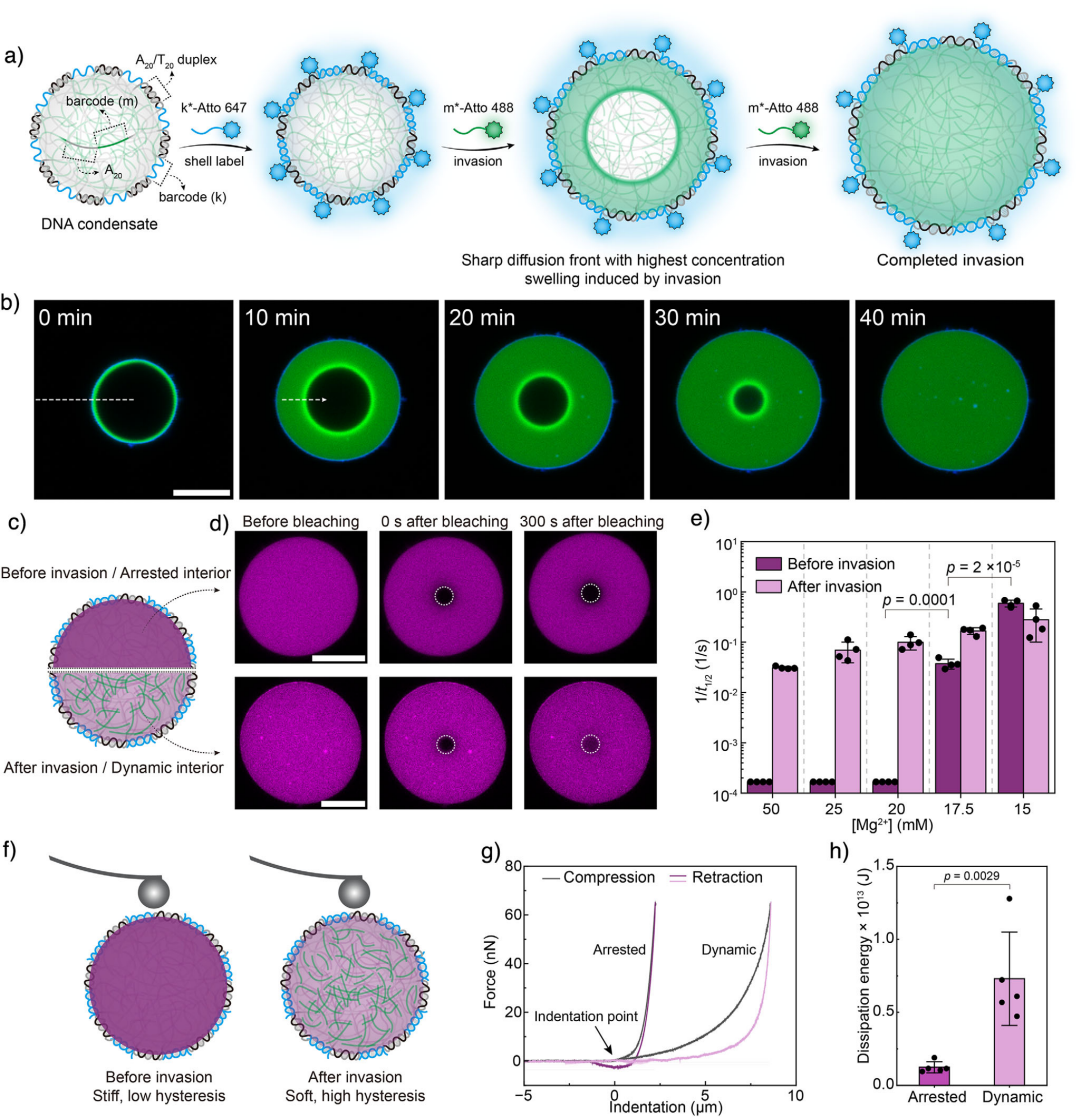
Arrested or dynamic properties of DNA condensates determined by matrix hybridization and salinity. a) Scheme for ballistic wave diffusion in a DNA condensate, which converts the condensate from an arrested to a dynamic state. b) Representative time‐series CLSM images showing the ballistic wave diffusion in a DNA condensate. c) Scheme for different dynamic properties of DNA condensate before and after invasion. d) FRAP experiments showing different recovery in bleached area (indicated by white dashed circle) before and after invasion. e) Reciprocal of half recovery time (1/*t*_1/2_) as a quantification for different dynamic properties of DNA condensates before and after invasion at various Mg^2+^ concentrations. f) Scheme for AFM force spectroscopy experiments on DNA condensates before and after invasion. g) Representative force spectroscopy in a full compression–retraction cycle of DNA condensates before and after invasion, showing different mechanical behaviors and hysteresis. h) Quantification of dissipation energy from hysteresis of force spectroscopy measurement of DNA condensates before and after invasion. Scale bars are 10 µm for (b) and (d). Figures are adapted from ref. [48] with permission under the terms of the Creative Commons CC BY license. Copyright 2025, The Authors.

Let us first focus on the simple influence of salinity on the condensate dynamics investigated by fluorescence recovery after photobleaching (FRAP) measurements. At high [Mg^2+^], condensate cores are dynamically arrested with no FRAP recovery, whereas lowering [Mg^2+^] below 17.5 mM yields fluid‐like condensates with substantial recovery (Figure 6c–e).^[^ ^48^ ^]^ This demonstrates how the persistence of percolation networks (Figure 4a,b) after cooling can be modulated.

An intriguing question arises: what happens when complementary ssDNA “invader strands” hybridize with the barcoded core of the condensate? This process revealed some real surprises. We discovered that invasion proceeds through an unprecedented transport mode deviating from classical Fickian diffusion.^[^ ^48^ ^]^ A sharp, high‐intensity front of invader strands propagates linearly with time while the condensate swells fourfold in volume (Figure 6a,b). We termed this behavior “ballistic wave diffusion.” This linear time dependence and accumulation of invader strand at the front is strikingly different from Fickian diffusion, characterized by a square‐root time dependence and blurred front. The cause for this “ballistic wave diffusion” is rooted in a critical transition of the condensate properties from an arrested to a dynamic state and the presence of defined supramolecular interaction between the invader and condensate core (barcode binding).

Upon invader binding, ssDNA converts to double‐stranded DNA (dsDNA), increasing polymer persistence length and causing local swelling that likely disrupts Mg^2+^‐mediated percolation bridges. FRAP measurements confirm this transition: before invasion, no recovery occurs, whereas after invasion, significant recovery indicates enhanced molecular mobility (Figure 6c,d). AFM force spectroscopy further supports this: pristine condensates show elastic, hysteresis‐free behavior typical of solids, whereas invaded condensates display viscous hysteresis, characteristic of liquid‐like materials (Figure 6f–h).

In a wider perspective, controlling condensate properties is crucial for constructing functional SCs. As we will discuss below, a liquid‐like, dynamic core promotes the assembly of DNA building blocks and supports enzymatic activity inside condensates. It also enhances material transport and biochemical conversion, both of which are crucial for creating functional SCs.

## 4 DNA Condensates for Various Applications

DNA condensates have taken the lead among nucleic acid condensates for systems chemistry, SC models, and application‐targeting research. From a DNA nanotechnology perspective, they represent a new class of mesoscopic DNA materials that extend beyond existing nanoscale design paradigms.^[^ ^8^, ^10^, ^14^, ^49^, ^50^, ^51^ ^]^ Their nucleic acid composition allows seamless integration with established DNA assembly strategies to create higher order superstructures, modulate internal architectures, and couple with enzymes or DNA reaction networks. Furthermore, their dense and macromolecularly crowded interiors (ca. 5–10 g/L) make them powerful SC models, complementing liposome systems that feature aqueous lumens.^[^ ^27^ ^]^ Moreover, the DNA‐rich interior provides a nucleus‐mimetic environment for studying transcription and PS phenomena. Ultimately, DNA condensates offer strong potential as life‐like materials capable of interacting and communicating with living cells. This section highlights their intersection with diverse research fields and their evolution as SC systems.

### 4.1 Integration with Colloid Science Approaches

**Figure 7:**
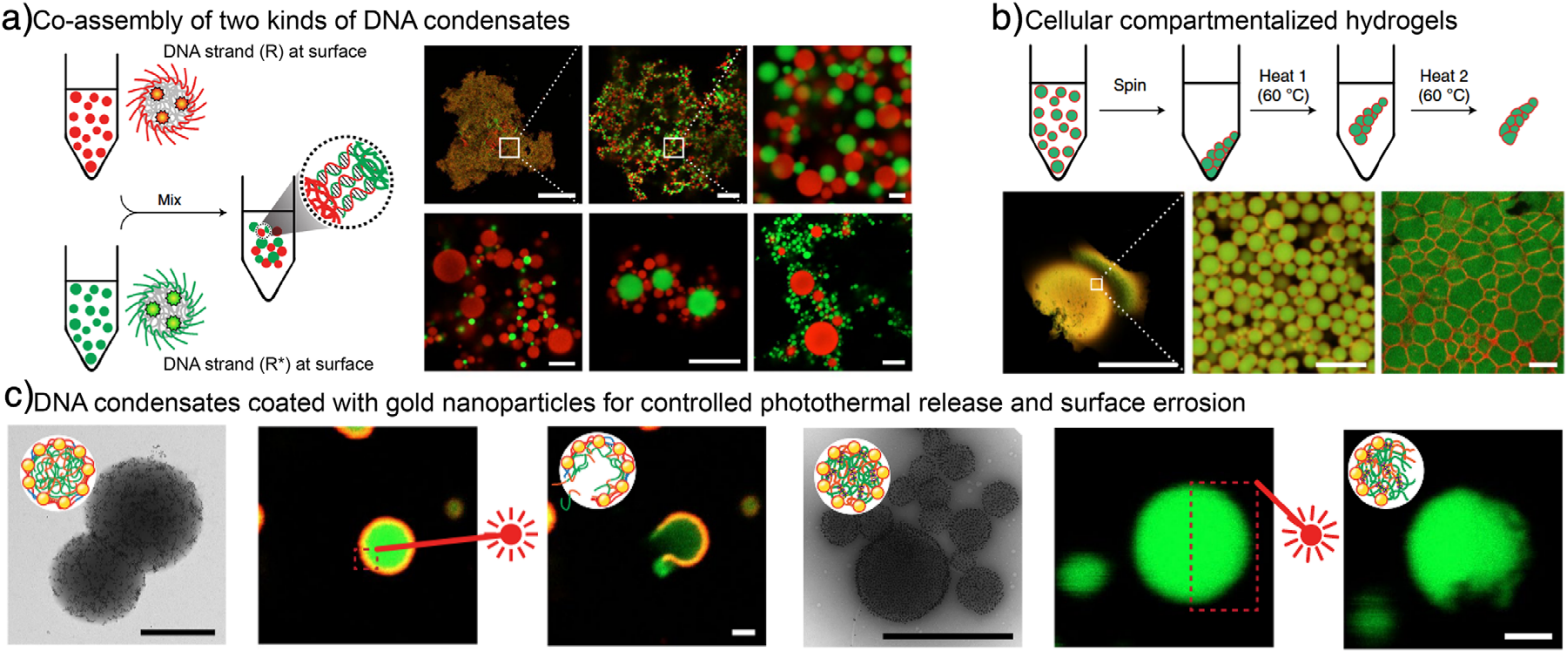
Formation of mesostructured DNA condensates and superstructures. a,b) Scheme and representative CLSM images showing the co‐assembly of condensates into a) colloidal assemblies, asymmetric core–satellite structures, and b) millimeter‐scale supra‐particulate hydrogels. c) TEM and CLSM images showing core–shell condensates and membraneless condensates coated with gold nanoparticles at the shell. Upon laser irradiation, core–shell condensates release the internal material as a result of shell breakage, whereas membraneless condensates only show selective erosion due to their arrested core. Scale bars are 200, 20, and 2 µm for (a) in different zoomed‐in images (left to right), 500, 5, and 5 µm for b) in different zoomed‐in images (left to right), and 1 µm for c). Figures are adapted from ref. [15] with permission. Copyright 2018, The Authors.

Owing to their all‐DNA composition, these condensates are naturally compatible with DNA nanotechnology, enabling sequence‐specific hybridization to form superstructures or incorporate DNA‐conjugated functional components. For example, we showed that two distinct condensate species (green and red) can co‐assemble into core–satellite architectures or proto‐tissues (Figure 7a).^[^ ^15^ ^]^ Moreover, centrifugation and reheating of core–shell condensates produced cell‐like compartmentalized hydrogels (Figure 7b). Finally, functionalizing condensates (both membraneless and core–shell types) with T_20_–HS–gold nanoparticles enabled photo‐thermal actuation, allowing spatiotemporal disruption of the shell and controlled release of liquid‐core contents (Figure 7c).

### 4.2 SCs with Artificial Secondary Structure

A key advantage of core–shell DNA condensates is their potential as novel SC models distinct from traditional liposomes or polymersomes.^[^ ^52^, ^53^, ^54^ ^]^ In short, DNA condensates offer programmable functionalization of both core and shell via barcode recognition, are permeable to large biomolecules, and possess a tunable, macromolecularly crowded interior that can emulate the dynamic and mechanical complexity of the cytoplasm.

**Figure 8:**
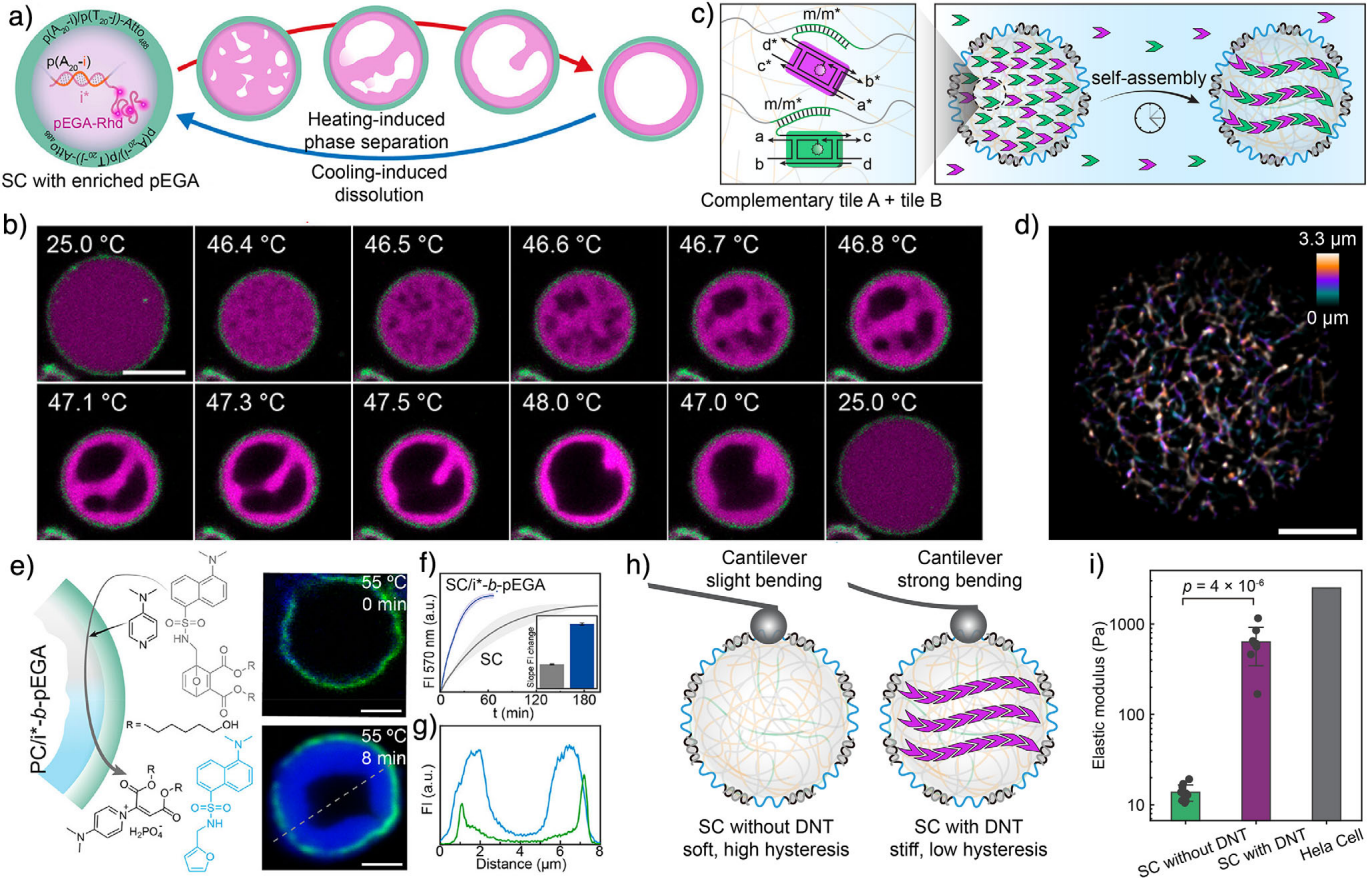
Formation of artificial substructures inside DNA for improved functionality and mechanical properties. a) Scheme of the thermo‐reversible PS of i*‐*b*‐pEGA in a SC for artificial organelle formation. b) CLSM images showing the thermo‐reversible PS of i*‐*b*‐pEGA (magenta channel) within an SC (green shell), following spinodal/viscoelastic PS. c) Scheme of the barcode‐mediated enrichment of DNA tiles and cytoskeleton formation in a SC. d) Structured illumination microscopy (SIM) image of artificial cytoskeletons formed in an SC. The color‐coded z‐projection image reveals branching morphology. e) Scheme and CLSM images showing the use of the hydrophobic subcompartments for promoting a retro‐Diels–Alder reaction. f) Time‐dependent fluorescence of the retro‐Diels–Alder reaction within SC/i*‐*b*‐pEGA hybrids versus normal SCs without i*‐*b*‐pEGA. g) Line segment analysis along the dashed line in (e). h) Scheme of the AFM force spectroscopy measurements on SCs with and without artificial cytoskeletons inside. i) Comparison of elastic modulus between SC with DNT, SC without DNT, and Hela cells. Scale bars are 5 µm for (b, d), and 2 µm for (e). Figures a, b, e–g are adapted from ref. [47] with permission. Copyright 2023, American Chemical Society. Figures c, d, h, i are adapted from ref. [27] with permission under the terms of the Creative Commons CC BY license. Copyright 2025, The Authors.

Exploring the structural complexity of cells raises the question of how artificial substructures, such as organelles or cytoskeletons, can form and function within the crowded confinement of DNA condensate SCs. To create switchable artificial organelles inside SCs, we embedded a thermo‐responsive ssDNA‐*b*‐polyethylene glycol acrylate (i*‐*b*‐pEGA) block copolymer via the i‐barcodes into the SC core.^[^ ^47^ ^]^ Upon heating, the pEGA block phase‐separated, evolving from a co‐continuous morphology to a concentric ring (Figure 8a,b). The resulting hydrophobic subcompartment served as a reaction crucible, enabling a retro‐Diels–Alder reaction between protected dansylfuran and 4‐Dimethylaminopyridine. The reaction, localized within the subcompartment, proceeded threefold faster than in pristine SCs, highlighting the benefit of responsive compartmentalization (Figure 8e–g).

For artificial cytoskeletons, we achieved in situ growth of DNA nanotubes (DNTs) within SCs by programming hybridization between DNT monomers and core barcodes (Figure 8c,d).^[^ ^27^ ^]^ Controlled enrichment of active barcodes within a passive matrix (Figure 5b–d) ensured ordered nucleation and branching, as revealed by super‐resolution microscopy (Figure 8d), which suggested a nonclassical growth mechanism via end‐to‐end joining of short fibrils rather than nucleation and growth as found in solution. Systematic variation of temperature, matrix crosslinking, and tile concentration demonstrated that condensate dynamics critically regulate cytoskeletal formation.^[^ ^27^ ^]^ AFM force spectroscopy further confirmed that cytoskeleton‐containing SCs display an elastic modulus two orders of magnitude higher than pristine SCs, approaching that of natural cells (Figure 8h,i).

### 4.3 SCs with Metabolic Functions

**Figure 9:**
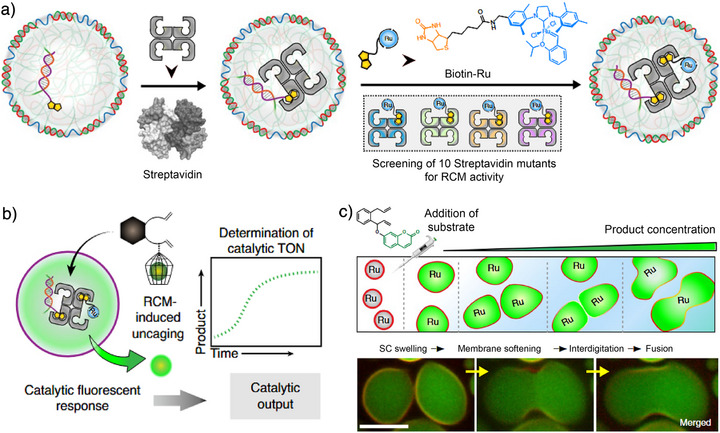
DNA SCs with encapsulated artificial metalloenzymes. a) Scheme for encapsulation strategy of artificial metalloenzyme into SCs by biotin–streptavidin interactions. b) The artificial metalloenzyme catalyzes a DNA‐orthogonal uncaging reaction by ring‐closing metathesis (RCM), yielding a primary fluorescent signal. c) Scheme and representative CLSM images of RCM‐induced morphological transformation and fusion of SCs. The scale bar is 5 µm for (c). Figures are adapted from ref. [23] with permission. Copyright 2020, The Authors, under exclusive license to Springer Nature Limited.

Beyond building complex internal structures, we have also created SCs with integrated metabolic functions. In a pioneering study, an artificial metalloenzyme was encapsulated via biotin–streptavidin interactions (Figure 9a).^[^ ^23^ ^]^ This conferred catalytic activity, enabling an uncaging reaction that produced a fluorescent reporter (umbelliferone) (Figure 9b). Owing to the DNA‐rich environment within the SCs, the artificial metalloenzyme exhibited enhanced catalytic efficiency compared to that in dilute solution, a result of macromolecular crowding. In addition, point mutations of the amino acid sequence, in particular relocating cationic amino acid residues, provided a boost in the catalytic efficiency. Remarkably, the product from the uncaging reaction (umbelliferone) induced a morphological response in SCs due to intercalation in the shell and subsequent membrane softening that allowed for swelling and finally fusion of neighboring SCs (Figure 9c). This study marks a significant advance in SC systems, demonstrating the ability to metabolize signals and undergo adaptive morphological changes.

**Figure 10:**
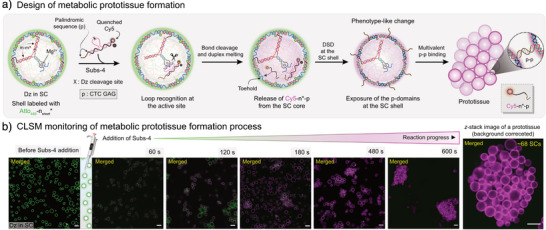
DNA SCs with encapsulated DNAzymes. a) Scheme for metabolic prototissue formation based on DNAzyme‐catalyzed substrate cleavage in SCs. b) Time‐series CLSM images showing the shell transformation and prototissue formation triggered by DNAzyme reaction in SCs. Scale bars are 5 µm for (b). Figures are adapted from ref. [25] under the terms of the Creative Commons CC BY license. Copyright 2022, The Authors.

In a complementary strategy, DNAzymes were encapsulated inside SCs via barcode‐mediated interactions, imparting catalytic activity to cleave specific DNA substrates containing RNA point modifications.^[^ ^25^ ^]^ As with artificial metalloenzymes, fluorescence assays showed enhanced activity when DNAzymes were confined within SCs. Further engineering of the reaction and system enabled strand production in the core, which subsequently translocated to the shell to trigger multivalent interactions, leading to signal‐mediated prototissue formation through encoded metabolic activity (Figure 10a,b).

Together, these strategies demonstrate the modular integration of metabolic functions into DNA‐based SCs. Looking ahead, such systems may evolve into fully modular SC platforms, where diverse structures and functions can be independently programmed, paving the way toward intelligent and multifunctional SCs.

### 4.4 Synthetic Protonucleus with Transcription Function

**Figure 11:**
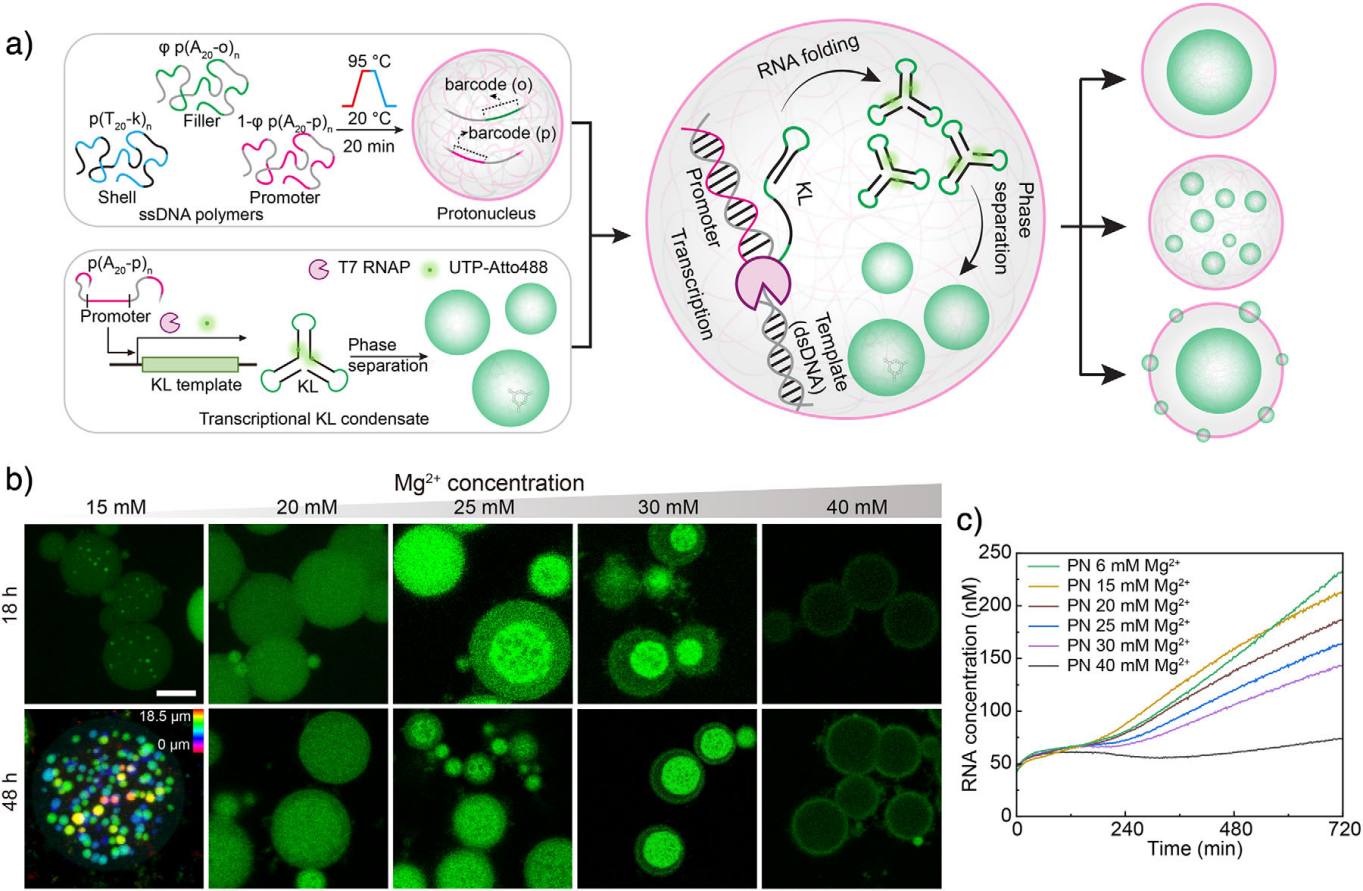
DNA condensates as protonuclei for transcriptional function and RNA condensation. a) Using co‐PS approach to form PN with embedded promoter sequence to gain transcription function. Transcription of RNA kissing loops (KLs) inside PN induces the formation of distinct nuclear patterns via different nucleation and condensation processes. b) Representative CLSM images showing the effects of [Mg^2+^] on transcriptional KL condensate formation in PN after 18 and 48 h reaction. c) Effects of [Mg^2+^] on transcription kinetics in PN, monitored via RNA‐triggered SDR of the reporter. Scale bar is 5 µm for (b). Figures are adapted from ref. [28] with permission under the terms of the Creative Commons CC BY license. Copyright 2025, The Authors.

Given their DNA‐rich interiors, it is natural to think about repurposing core–shell DNA condensates as nucleus‐mimicking compartments or protonucleus (PN) to study nuclear processes such as DNA replication, RNA transcription, and PS in environments reminiscent of the cell nucleus. To introduce transcriptional functionality, a T7 RNA polymerase (T7 RNAP) promoter sequence was embedded as a barcode in the SC core (Figure 11a).^[^ ^28^ ^]^ Supplying the corresponding template strand enabled in situ transcription within SCs. Using light‐up aptamers and fluorescent nucleotides, CLSM imaging confirmed localized transcription activity inside the PN, with transcript levels comparable to bulk solution.^[^ ^28^ ^]^ Even more intriguingly, when a DNA template encoding RNA kissing‐loop (KL) motifs (i.e., intermolecular binding segments) is used,^[^ ^39^, ^40^ ^]^ the transcribed RNA spontaneously forms transcriptional condensates within the PN. Because Mg^2+^ ions modulate both transcription rate and KL binding strength, varying [Mg^2+^] produces distinct RNA‐condensate morphologies (Figure 11b,c). At low [Mg^2+^], faster transcription yields many small condensates from widespread nucleation, whereas high [Mg^2+^] slows transcription and strengthens interactions, favoring a single large condensate through co‐continuous PS and compaction. This study underscores the versatility of DNA condensates as synthetic platforms for exploring nuclear processes and functions in nucleus‐mimetic environments.

### 4.5 SC‐Natural Cell Interface

**Figure 12:**
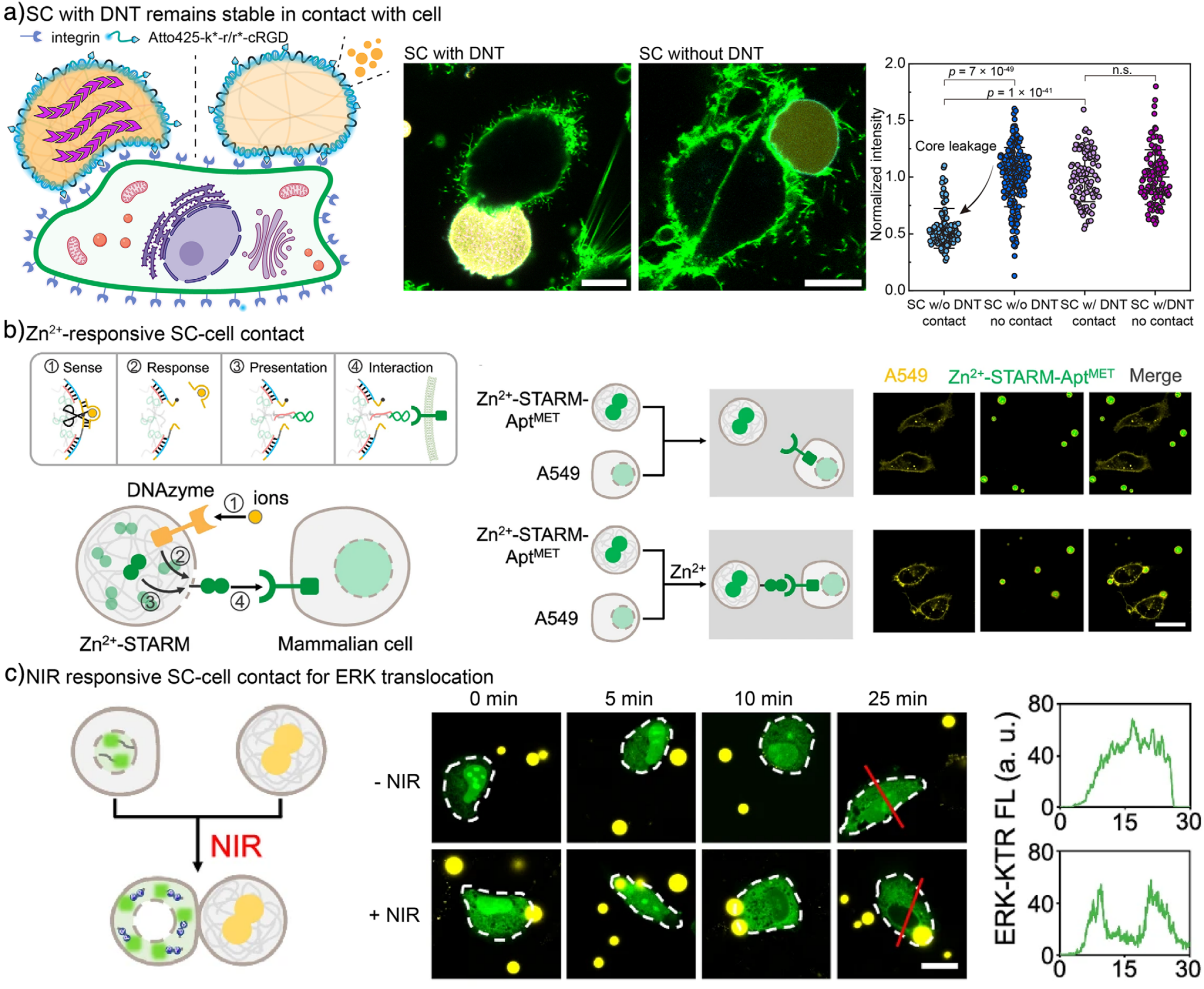
Construction of SC‐cell interacting interface. a) Scheme and CLSM images showing the SC–cell contact mediated by cRGD–integrin binding. The artificial cytoskeletons provide mechanical stability to SCs, ensuring a durable SC–cell interface. In the absence of the internal cytoskeleton, significant leakage of liquid SC core material occurs. b) Scheme and CLSM images showing the SC–A549 cell interaction through Zn^2+^‐mediated activation of DNAzyme, which cleaves the SC shell and releases internal aptamer for cellular receptor binding. In the absence of Zn^2+^, SC–A549 cell interaction cannot be observed. c) Scheme and CLSM images showing NIR‐triggered SC–A549 cell interaction, leading to ERK translocation. Scale bars are 10 µm for (a) and 20 µm for (b, c). Figure a is adapted from ref. [27] with permission under the terms of the Creative Commons CC BY license. Copyright 2025, The Authors. Figures b, c are adapted from ref. [29] with permission under the terms of the Creative Commons CC‐BY‐NC‐ND license. Copyright 2025, The Authors.

One of the most promising and challenging directions for DNA‐based SCs is the creation of interfaces with natural cells to enable targeted interaction, communication, and cellular regulation. Although still in early development, two notable strategies have emerged. First, we explored how the enhanced mechanical stability of cytoskeleton‐containing SCs supports an SC–cell mechano‐interface. Functionalizing the SC shell with cyclic Arg–Gly–Asp (cRGD) peptides, which bind integrin receptors on cells, enabled specific adhesion (Figure 12a).^[^ ^27^ ^]^ Upon contact, the internal cytoskeleton provided sufficient mechanical integrity to withstand cellular forces and prevent rupture (Figure 12a).

Second, nucleic acid aptamers can be employed to target specific cell‐surface receptors and modulate cellular functions. In a recent study, Nie and coworkers developed DNA‐based SCs responsive to Zn^2+^ and photo‐thermal heating.^[^ ^29^ ^]^ Zn^2+^ activation triggered a Zn^2+^‐responsive DNAzyme that cleaved the SC shell, exposing MET‐targeting aptamers in the core (MET = Mesenchymal‐Epithelial Transition), which then bind to MET receptors on A549 cells (Figure 12b). Incorporating gold nanorods enabled near‐infrared (NIR) irradiation to locally heat and disrupt the shell, similarly exposing MET‐targeting aptamers from SCs to interact with A549 cells. This induced downstream signaling, such as ERK (extracellular signal‐regulated kinases) translocation from the nucleus to the cytoplasm (Figure 12c). Remarkably, Zn^2+^‐ and NIR‐triggered aptamer release can function independently and orthogonally, allowing selective ERK regulation in distinct cell types.

Both studies underscore the potential to expand the DNA‐based SC platform toward advanced communication networks. By co‐phase‐separating multiple barcoded ssDNA polymers and assigning distinct functions to individual barcodes, it becomes conceivable to build multifunctional SCs capable of executing complex tasks and programmable interactions with living systems.

## 5 Conclusions and Outlook

Temperature‐induced PS of nucleic acids has opened a new frontier at the intersection of nucleic acid nanoscience, soft matter, systems chemistry, as well as synthetic and cell biology. Beyond traditional DNA nanotechnology, long DNA and RNA polymers display LCST‐type condensation, revealing how sequence composition, ion identity, and molecular weight dictate macroscopic phase behavior. In particular, RNA PS provides new insight into the physical underpinnings of biological condensates and offers an experimentally tractable model for studying sequence‐encoded self‐organization in gene regulation and prebiotic evolution.

Harnessing these principles has enabled the creation of DNA condensates as programmable, life‐like materials that combine compartmentalization, metabolism, and information flow. Barcode‐mediated recognition, crosslinking motifs, and coupling to DNA or RNA reaction networks now allow SCs to sense, compute, and actuate in response to molecular cues, laying the groundwork for chemical information processing and distributed communication between synthetic and natural systems. At the same time, new programmable model systems for the cytoplasm or even the nucleus have become available to study structure formation and dynamic processes outside of cells.

Looking ahead, challenges lie in deepening the molecular understanding of PS and in elucidating the sequence rules governing DNA/RNA condensation. Integrating DNA condensates with protein machineries, signal transduction, feedback regulation, and energy‐providing metabolism may ultimately yield adaptive, computing SC entities capable of evolutionary learning and interactive behavior.

Thus, the temperature‐induced PS of nucleic acids not only expands the scope of DNA and RNA nanotechnology but also provides a conceptual bridge toward SCs and embodied molecular intelligence, advancing the vision of programmable matter approaching life.

## Conflict of Interests

The authors declare no conflicts of interest.

## Data Availability

Data sharing is not applicable to this article as no new data were created or analyzed in this study.
